# A Preliminary Study of a Prototype Cryoablation Needle on Porcine Livers for Pancreatic Cancer Treatment

**DOI:** 10.3390/jcm13174998

**Published:** 2024-08-23

**Authors:** Hyunjoon Son, Tae In Kim, Jonghyun Lee, Sung Yong Han, Dong Uk Kim, Daejin Kim, Gun-Ho Kim

**Affiliations:** 1 Department of Mechanical Engineering, Ulsan National Institute of Science and Technology, Ulsan 44919, Republic of Korea; hjson1317@unist.ac.kr (H.S.); daejin0119@unist.ac.kr (D.K.); 2 Division of Gastroenterology, Department of Internal Medicine and Biomedical Research Institute, Pusan National University Hospital, Busan 49241, Republic of Korea; zeitgeister88@daum.net (T.I.K.); keiasikr@nate.com (J.L.); 3 Internal Medicine, School of Medicine, Pusan National University, Busan 46241, Republic of Korea; 4 Division of Gastroenterology, Department of Internal Medicine, CHA Gumi Medical Center, CHA University, Gumi 13488, Republic of Korea; amlm3@hanmail.net

**Keywords:** pancreatic cancer, cyroablation, endoscopic ultrasonography

## Abstract

**Background and Aims:** Despite its relatively low incidence rate compared to others, pancreatic cancer has a poor prognosis owing to its late detection and poor response to systemic chemotherapy. Because the effectiveness of chemotherapy is still restricted, the need for locoregional treatment is increasing. Cryoablation is an effective and minimally invasive treatment for some cancers, but its efficiency in pancreatic cancer is limited. Despite recent reports about promising outcomes, the optimal method and conditions of treatment are not known. In this preliminary study, we aimed to develop a cryoablation needle which can control the ablated area considering application through endoscopic ultrasonography. **Methods:** Here, we used a novel cryoneedle cooling system which can adjust the ablation range based on a liquid carbon dioxide refrigerant. Applied to the livers of swine, the cryoablation needle rapidly reached −60 °C within 30 s and cryoablation was performed for approximately 240 s. Based on the distance and depth, we collected real-time temperature data during the procedure. To compare the extent of cell death over time, tissue samples were collected hourly from 3 to 6 h after the procedure. **Results:** Approximately 4–5 mm of tissue was ablated via cryoablation, and cell death progressed over time after cryoablation. Moreover, the ablated lesions could be regulated using an insulating agent on the needle. **Conclusions:** This preliminary study on a novel surgical cooling needle system compatible with endoscopic ultrasound for cryoablation-based pancreatic cancer treatment confirmed the efficacy of cryoablation and identified the conditions necessary to induce necrosis. Additionally, this study evaluated the effectiveness of the insulation component of the system in protecting normal cells and assessed the extent of necrosis over time after the procedure.

## 1. Introduction

Pancreatic adenocarcinoma was the fourth leading cause of cancer-related mortality in the United States in 2020. It has a poor prognosis, with a 5-year survival rate of 6% in Europe and the United States [1,2]. Surgery is essential for cure; however, only 5–25% of cases are operable at the time of diagnosis. Even with surgery, the 5-year survival rate is under 50% for this disease [3]. Since 2010, the 5-fluorouracil, irinotecan, and oxaliplatin (FOLFIRINOX) regimen has been the mainstay chemotherapy for patients with metastatic pancreatic cancer. However, the proportion of patients achieving complete remission or becoming operable with chemotherapy alone is very low, necessitating the involvement of alternative cytotoxic chemotherapy, namely immune checkpoint inhibitors (ICIs) as a palliative treatment. However, these have not been extensively investigated and their efficacy remains unknown. Currently, other treatment methods, such as radiofrequency ablation (RFA) [4], microwave ablation (MWA) [5], irreversible electroporation (IRE) [6], and cryoablation [7], are being tested to improve patient survival in clinical trials. RFA utilizes alternating electrical currents to create a thermal ablation zone in the tissue. MWA creates an ablation zone by generating frictional heat through the agitation of water molecules. They are inexpensive and widely available compared to other ablation modalities, but serious adverse events include pancreatic fistula, acute pancreatitis, bleeding, and liver failure, which can be fatal [8].

Cryoablation is commonly used to treat skin and cervical cancers, and many ongoing studies are investigating its effects on kidney, liver, lung, and prostate cancers. Its effects on cryoablation on pancreatic cancer have also been investigated [9,10]. Some types of tools like the cryoballoon or needle have been used for cryoablation, but a major drawback of the cryo-balloon is the inability to cool only the surface of the lesion. And most commercially available cryoneedles ablate too large an area, potentially damaging proximal normal tissue besides the target lesion located in a deep portion of the pancreas, increasing the risk of complications. Also, some studies have shown the promising results of cryoablation therapy in patients with advanced pancreatic cancer; however, most studies are generally performed intraoperatively or percutaneously, thereby increasing the labor, cost and risk of the adverse events associated with surgery [11]. The efficacy of cryoablation for pancreatic cancer treatment can be improved using endoscopic ultrasound (EUS) to collect tissues for diagnosis. Therefore, in this preliminary study, we aimed to develop a novel cryoablation needle for EUS and to precisely control the ablated region with the aim of reducing complications. Here, we developed a prototype cryoablation system to evaluate the degree of cell death induced by cryoablation in porcine livers depending on the ablated temperature and time. 

## 2. Methods

This experiment was conducted to determine the cooling temperature and time conditions required for necrosis of liver cells in swine and to evaluate the protective effect of insulation on normal cells and the range of cryoablation. This study involved two swine which were anesthetized and underwent laparotomy. The experiment was carried out using the cooling needle for the cryoablation, temperature sensors for real-time temperature measurements, and a jig to secure the sensor’s position. Additionally, to assess the progression of cell death over time, cryoablation procedures were performed at consistent time intervals on different surgical sites, and samples were collected to compare the extent of cell death.

### 2.1. Cryoablation System

**Figure 1:**
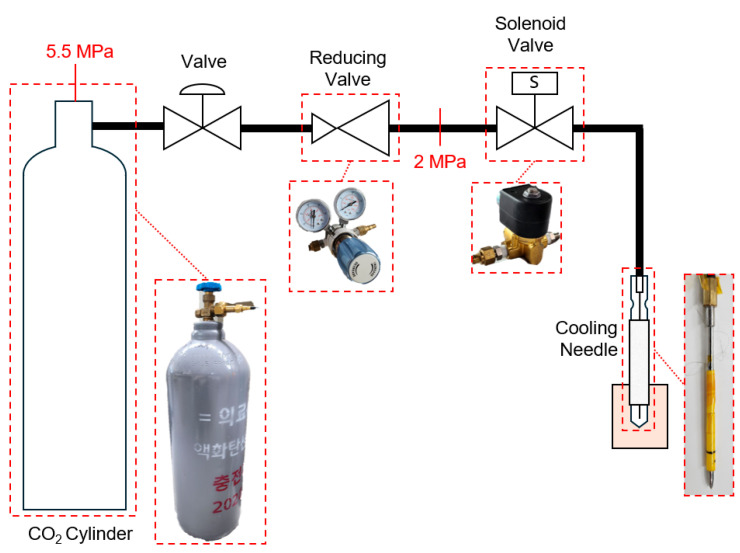
Schematic diagram of cryoablation system. Non-English sentence is meaning for “Medical liquid carbon dioxide”.

The cooling system of the cryoablation device was designed based on a liquid carbon dioxide refrigerant exhibiting a higher Joule–Thomson coefficient than the other refrigerants at the same temperature. This characteristic facilitates a significant temperature drop relative to the same pressure difference. This system was configured with a cooling needle system and refrigerant control system (Figure 1). The cooling needle system consists of a cooling needle that pierces the target and cools the target point, a cooling nozzle that sprays the refrigerant onto the needle, and an insulating wall that prevents heat transfer beyond the target area from the outer wall of the cooling needle, thereby preventing undesired ablation in normal cells.

**Figure 2:**
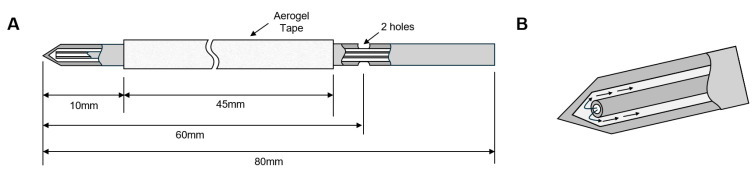
(**A**) Configuration of cryoneedle. (**B**) Action mechanism of cryoneedle.

The cooling needle, with a 3 mm outer diameter, an 80 mm length, and a 60° angled tip to facilitate easy insertion into the target, was inserted into an endoscope, reaching the target with minimal force. To ensure efficient heat exchange with the target and the proper discharge of carbon dioxide, the inner diameter was set as 2 mm. Two 1.5 mm holes were placed approximately 60 mm from the tip for safe refrigerant discharge. The cooling nozzle, positioned inside the cooling needle, was constructed with a pipe shape with a 1 mm outer diameter, a 0.7 mm inner diameter, and a 78 mm length (Figure 2). It generated the Joule–Thomson effect through a 0.4 mm diameter orifice, cooling the needle to approximately −60 °C. The insulating wall, with a thickness of approximately 0.5 mm and thermal conductivity of 0.036 W/m·K, is made of an aerogel tape (roVa Flex Plus, roVa) and installed at 10 mm after the tip of the cooling needle and just before the outlet.

The refrigerant control system comprises a supply unit, a reducing valve (Model SR4B; Dae-a Machinery & Electric Co., Ltd., Daegu, Republic of Korea) that controls the pressure of the supplied refrigerant to achieve the desired pressure, and a solenoid valve (Model C322C1; Parker, Richland, MI, USA) controlled by a control board to regulate the supply time and interval of the refrigerant. The reducing valve reduces the pressure from the initial 55 bar of the source to the target procedural pressures of 30 and 20 bar, while simultaneously cooling the refrigerant to subzero temperatures due to the Joule–Thomson effect. This contributes to more effective cooling when the refrigerant is expelled through the nozzle. The operating conditions were as follows: pre-cooling at 30 bar for the first 50 s under in vivo conditions, depressurization at 20 bar for 210 s, and removal of the needle after standing for 60 s to facilitate the melting of the tissue around the cooling needle.

### 2.2. Temperature Measurement

**Figure 3:**
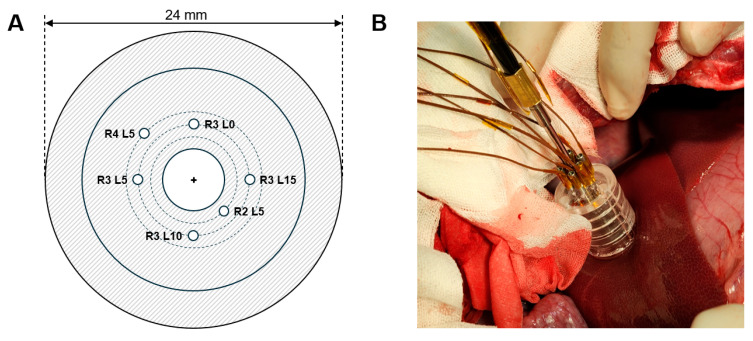
(**A**) A schematic diagram of the acrylic jig. (**B**) A surgical photograph.

During cryoablation surgery, the temperature of the cooling needle was measured by T-type thermocouples with a diameter of 70 μm. To monitor the temperature in each section of the cooling needle, the thermocouples were positioned 5, 10, and 15 mm away from the tip. To monitor the temperature distribution based on the cooling performance of the cooling system and the distance from the cooling needle to the treatment target, 22G SUS304 needle thermocouples were inserted into the target for temperature measurement. These temperature sensors were positioned at the target measurement points based on the difference in length between each needle and acrylic jig (Figure 3A). These measurement points were configured as combinations of radial distances (0 [R0], 2 [R2], 3 [R3], and 4 [R4] mm) from the surface of the cooling needle and distances (0 [L0], 5 [L5], 10 [L10], and 15 [L15] mm) from the tip of the cooling needle (Figure 3B). Finally, thentemperature data for each interval measured over time were compared with the area of deceased cells post-procedure to verify the temperature and cooling time required for the treatment.

### 2.3. Procedure

This study was approved by the Institutional Animal Care and Use Committee (No. P2023-005-A1C0). Approximately 30 kg of swine tissue was used for cryoablation. Before the procedure, an incision was made in the midline of the abdomen, and the liver was exposed as much as possible. A needle was inserted >2 cm into the swine liver. The first swine was subjected to cryoablation twice and the second swine was subjected to a fourth cryoablation at different sites in each procedure. The livers were collected from the swine and fixed in formalin. After liver sectioning, hematoxylin and eosin (H&E) staining was performed to evaluate the degree of liver tissue damage. The progression of cell death over time was examined by collecting tissue samples from the swine every 1 h from 3 to 6 h after ablation.

To determine the size of necrosis in a living animal at a specific temperature, we needed to apply the needle on an organ as large as possible. Because the size of a porcine pancreas is relatively small, we thought that it could be hard to measure the range of necrosis precisely from ablation. Additionally, porcine livers are not difficult to access. These advantages led us to select the liver of the swine for the experiments.

### 2.4. Necrosis

Cell necrosis is defined as the loss of plasma membrane integrity. Morphologically, necrosis occurs in several forms [12]. The pathological appearance after cryoablation includes central necrosis (amorphous material and cellular debris) and an inflammatory wall (granulation tissue with fibroblastic reaction, new blood vessels, and many lymphocytes and polymorphonucleated neutrophil granulocytes) [13]. After the final procedure, the swine were euthanized within 30 min, and we extracted and preserved their livers in formalin. To assess the extent of liver tissue damage, the livers were sectioned and stained with hematoxylin and eosin. The extent of liver necrosis was evaluated using the Batts–Ludwig system. Necrosis, or cell death, involves the transition of viable cells to a nonviable state, leading to the dissolution of cell contents. Scattered necrosis was classified as mild, necrotic clusters as moderate, and prominent diffuse damage as severe.

## 3. Results

### 3.1. Temperature of Needle and Surrounding Tissue during Cryoablation

**Figure 4:**
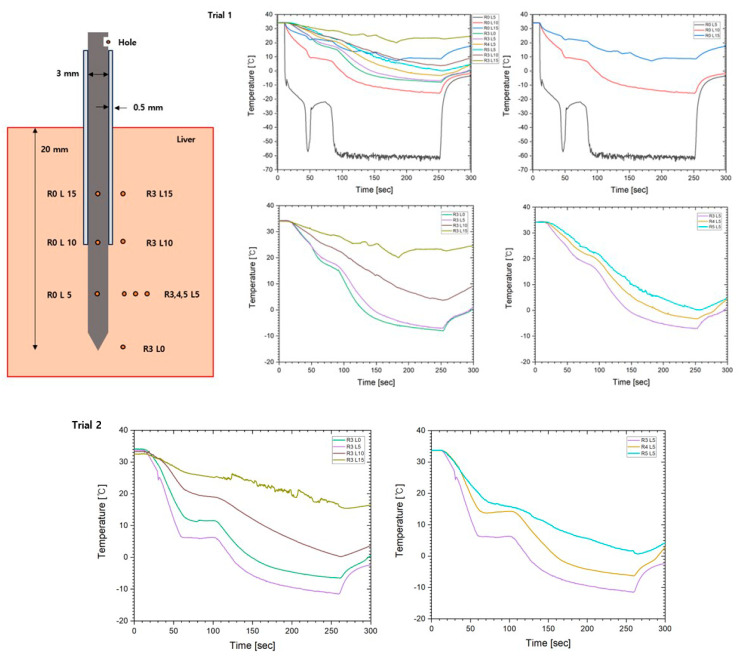
A schematic image of the cryoablation needle and the temperature curves of trials 1 and 2.

Figure 4 shows a schematic diagram of the cryoablation needle and the temperature curve of the needle and the surrounding tissue during the procedure. Trial 1 and 2 were performed under the same conditions, with only the locations changed on the first swine. The thermocouples at the needle did not work after the first trial; therefore, the needle temperature was not measured in the second trial. R0 L5 showed a drop below −60 °C within 30 s of the start of the procedure and had the lowest temperature in the needle. In a prior study, R0 L10 dropped below −50 °C; however, the temperature was measured at −10 °C due to the effect of insulation. R0 L15 was measured over 10 °C, which was also related to the insulation effect. The left middle and bottom graphs show that each trial exhibited a high temperature at 3 mm (R3) from the needle area, which was 0, 5, 10, and 15 mm (L0, L5, L10, and L15, respectively) from the tip of the cooling needle. The temperature of the L0, L5, and L10 areas dropped to −10, from −10 to −5, and from 0 to 5 °C, respectively. R3 L0 and R3 L5 dropped below 0 °C; however, R3 L10 did not reach below 0 °C in trials 1 and 2. The right middle and bottom graphs show that each trial exhibited high temperatures at 3, 4, and 5 mm (R3, R4, and R5) from the 5 mm (L5) needle tip. The temperature of the R3, R4, and R5 areas dropped from −15 to −10, from −5 to 0, and from 0 to 5 °C, respectively. R3 L5 and R4 L5 dropped below 0 °C; however, R5 L5 did not reach below 0 °C in trials 1 and 2. 

### 3.2. Effects of Cryoablation on Swine Liver

**Figure 5:**
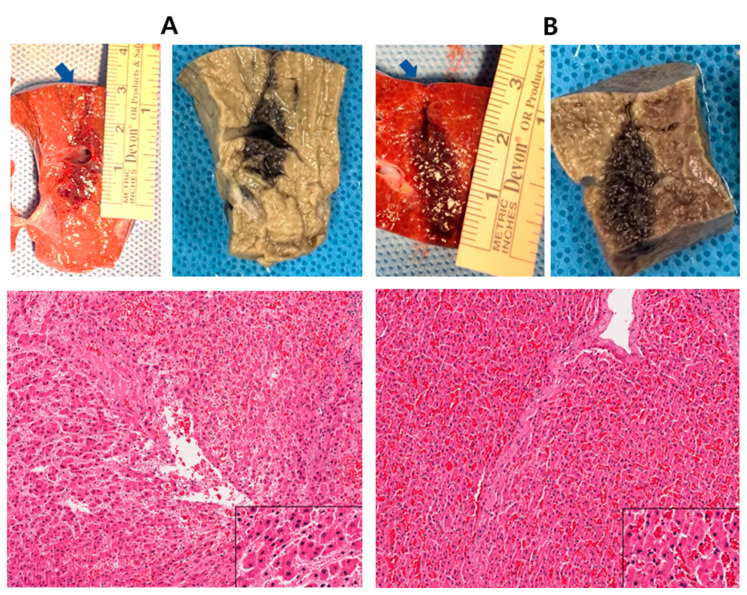
(**A**) #1 swine: cryoablation was performed 3 h before sacrifice (×10 hematoxylin and eosin [H&E] stain). (**B**) #1 swine: cryoablation was performed 30 min before sacrifice (×10 H&E stain). Blue arrows mean inserted sites of cryoneedle.

Cryoablation was performed 3 h before animal sacrifice, as shown in Figure 5A, and 30 min before animal sacrifice, as shown in Figure 2B. The first picture shows the swine liver after sacrifice, and the second picture shows the liver fixed with formalin (Figure 5A,B). The ablation area had a long diameter of 2 cm and a short diameter of 1 cm. As the effective ablation area of the needle was 1 cm in length and 3 mm in diameter, approximately 4–5 mm of the tissue was ablated. The third image shows the H&E-stained slide (×10 H&E stain). Figure 5A shows moderate cell death, and Figure 5B shows relatively intact cells with hemorrhage.

**Figure 6:**
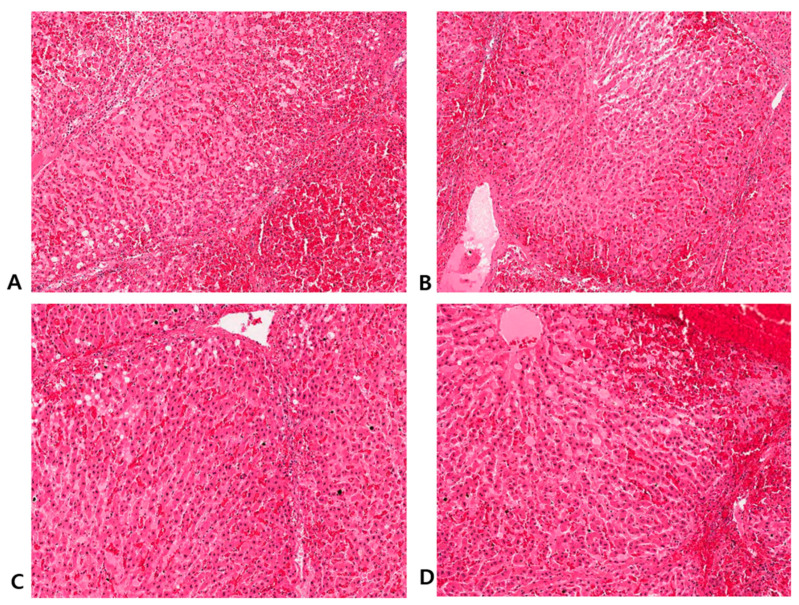
(**A**) #2 swine: cryoablation was performed 6 h before sacrifice (×10 H&E stain). (**B**) #2 swine: cryoablation was performed 5 h before sacrifice (×10 H&E stain). (**C**) #2 swine: cryoablation was performed 4 h before sacrifice (×10 H&E stain). (**D**) #2 swine: cryoablation was performed 3 h before sacrifice (×10 H&E stain).

Figure 6 shows H&E staining at various time points after the procedure. A (6 h), B (5 h), C (4 h), and D (3 h) show tissues ablated under the same conditions; cell death progressed over time, and the structure between the cells gradually collapsed.

## 4. Discussion

In this study, the cryoablation system developed by our team was applied to the liver of swine. Cell temperature was measured during the procedure, and the degree of cell necrosis was measured after the procedure. The thermocouple malfunctioned after the first trial; therefore, the needle temperature was not measured during the second trial. The temperature at R0 L5 reached a minimum of −60 °C within approximately 50 s after the initiation of the procedure, whereas that at R0 L10 was −10 °C and that at R0 L15 was >10 °C due to the insulation effect. The cryoablation needle rapidly reached −60 °C, and cryoablation was performed for approximately 240 s. This confirmed the occurrence of necrosis in the swine liver. As the effective ablation area of the needle was 1 cm in length and 3 mm in diameter, approximately 4–5 mm of the tissue was ablated. In other words, when comparing the ablation range of these cells with the procedural conditions, it can be concluded that necrosis occurs when liver cells are maintained below 0 °C for approximately 100 s.

A previous version of this cryoneedle attained temperatures below −40 °C along its entire length. This may inadvertently cause cell death in normal tissues around the cancerous lesions, particularly if the cancer cells are located deep within the organ. Therefore, in this study, an insulator was incorporated into a section of the needle to control the degree of cryoablation. In contrast to commercial cryoneedles, such as the HybridTherm Probe (ERBE Elektromedizin GmbH, Tübingen, Germany), that only cool a portion of the needle, our novel cryoneedle cooled the entire section of the needle by insulating specific areas. As shown in Figure 5, ablation occurred in a club-shaped pattern compared to that in the section with the insulator. However, cryoablation is not widely used due to the high risk of damage to other organs owing to the small volume and fragile parenchyma of the pancreatic gland and its proximity to the duodenum, colon, and common bile duct [9]. Therefore, the use of an insulator can minimize the damage to normal cells and facilitate cryoablation in the desired region. 

Figure 6 shows that the cell structure collapsed and cell death progressed over time after cryoablation. This may be because cryoablation induces cell necrosis through a mechanism different from that of other localized treatments. Cryoablation induces cell death via direct and indirect mechanisms. As the cryo-probe absorbs heat, ice crystals form in the extracellular space, trapping free water and increasing the osmotic pressure in the extracellular space [14], resulting in damage to cytoplasmic enzymes and instability of the cell membrane. However, peptide bonds are not broken, and protein denaturation can be reversed through heating or rehydration. However, if the tissue is cooled rapidly, there is insufficient time for intracellular dehydration and free water becomes trapped inside the cells. This rapid cooling causes intracellular ice crystal formation, leading to immediate cell death [15,16]. Moreover, cells that are not destroyed by the direct mechanism undergo apoptosis or programmed cell death involving cysteine-aspartate protease (caspase) [17]. This may be the mechanism involved in delayed cell death that ensures active cell death at the periphery of the cryoablation zone [18]. 

The development of systemic chemotherapy has increased the treatment options available for unresectable pancreatic cancer. However, the only cure is complete resection, and the 5-year survival rate for metastatic cases is approximately 10% [19]. Conversion surgery is performed after systemic chemotherapy for metastatic or locally advanced pancreatic cancer. However, the recurrence rate after surgery is 80%; therefore, palliative adjuvant therapies, such as radiation therapy and radiofrequency ablation, are also performed [20]. Cryoablation is a promising option for these cases. Although the research is limited, cryoablation has yielded positive results in many other cancer types. In vitro studies have demonstrated that a single exposure to −25 °C induces irreversible cell death in PANC-1 (pancreatic cancer) cells. Repeated cryoablation cycles achieve similar effects at higher temperatures [21,22]. The key issue limiting its widespread use is the ease of application of cryoablation. Wu et al. reported the efficacy and safety of laparoscopic ultrasonography-guided cryoablation for locally advanced pancreatic cancer [23]; however, it is not widely used. The use of cryoablation needles with insulated sections for pancreatic cancer using EUS can be a good alternative with high safety and efficacy. Carrara et al. reported a case–control study in which cryoablation was performed in patients with pancreatic cancer using EUS [24]. The cryotherm probe (CTP) used in the study is basically used for radiofrequency ablation. An existing ERBOKRYO CA system was used to spray CO_2_ gas through a sharp tip, which induced cooling. Its Joule–Thomson-based gas systems are quick but cannot generate a proper cooling effect for some purposes, such as endoscopic treatment of the pancreas. Baust et al. conducted a preliminary study on a novel endoscopic EUS cryocatheter for ablating pancreatic cancer [25]. The cryocatheter used in that study, FrostBite (Phase Tx, Owego, NY, USA), had a diameter of 8 Fr (2.6 mm), making it compatible with EUS scopes; mixed-phase nitrogen was used as a cryogen. Nitrogen systems produce very cold temperatures, but they are slow and require the use of large probes. In contrast, liquid CO_2_ used in this study was delivered as a two-phase jet when sprayed onto the tissue, resulting in a higher heat exchange efficiency. This, along with a higher Joule–Thomson coefficient, allows for more precise control of the temperature and area of cryoablation. Our cryoneedle with thermal insulation facilitated the maintenance of a low temperature only for the needle in the target area, allowing for adjustments to absorb more heat.

This study has several limitations. Technically, it should be addressed to facilitate the application of the developed cryoablation device to actual endoscopic equipment for surgery. The temperature of the refrigerant discharged to the digestive system through the outlet is as low as 2 °C, which may not cause serious damage like at 0 °C but may cause temporary or unintended damage to the stomach or digestive system. Therefore, increasing the temperature of the discharged refrigerant or supplementing the system such that the refrigerant can be discharged from the body is necessary for effective treatment. Moreover, this system was designed to treat pancreatic cancer by inserting the endoscope into the stomach. To insert a cryoneedle through the endoscope, it is essential to reduce the diameter of the hose-coupling section to less than 3.7 mm. However, our cryoneedle system has a diameter of approximately 4 mm, including the insulator; therefore, it cannot be inserted into the channel of the endoscope in its current form, thereby necessitating an additional system configuration. Additionally, delayed adverse events occurring after cryoablation need to be further investigated. Cryoablation exhibits a lower risk of side effects from thermal damage compared to heat-based thermal ablation techniques, such as microwave or radiofrequency ablation; however, abscesses may be formed due to cell necrosis and bioenteric infection. The swine used in this study were sacrificed up to 6 h after cryoablation; however, abscess formation takes at least a few days. Therefore, the time in this case may not be sufficient to determine the relationship between cryoablation and abscess formation. To overcome this issue, we plan to conduct additional experiments in swine sacrificed 3–5 d after ablation. Finally, this study only focused on the effects of cryoablation on normal liver tissues of swine. However, further research is necessary to determine its effects on pancreatic tissues. The stromal component of pancreatic cancer cells is characterized by a lack of vascularity, which further impedes the delivery of therapeutic agents. High vascularity is related to the malignant phenotypes of several types of cancers [26,27], and the efficacy of cryoablation in pancreatic tumors with relatively low vascularity requires further experimental and clinical validation.

Overall, our findings suggest cryoablation as a promising treatment option for patients with pancreatic cancer. Here, we found that cryoablation in pig livers using a specially designed cryoneedle led to increased cell death that progressed over time. We also confirmed that the ablation range was intentionally controlled by an insulation section, resulting in a reduced area of cell death which facilitates precise and targeted treatment. However, further research is necessary to develop cryoneedles for other endoscopic procedures.

## Data Availability

The original contributions presented in the study are included in the article, further inquiries can be directed to the corresponding authors.
